# Comparative- and Cost-Effectiveness Research Determining the Optimal Intervention for Advancing Transgender Women With HIV to Full Viral Suppression (Text Me, Alexis!): Protocol for a Randomized Controlled Trial

**DOI:** 10.2196/65313

**Published:** 2025-01-23

**Authors:** Cathy J Reback, Thomas Blue, Ali Jalali, Raphael Landovitz, Michael J Li, Raymond P Mata, Danielle Ryan, Philip J Jeng, Sean M Murphy

**Affiliations:** 1 Friends Research Institute, Inc. Baltimore, MD United States; 2 Department of Family Medicine, University of California Center for HIV Identification, Prevention and Treatment Services Los Angeles, CA United States; 3 Department of Family Medicine Center for Behavioral and Addiction Medicine Los Angeles, CA United States; 4 Department of Population Health Sciences Weill Cornell Medicine New York, NY United States; 5 Center for Health Economics of Treatment Interventions for Substance Use Disorder, HCV, and HIV New York, NY United States; 6 Division of Infectious Diseases University of California Los Angeles, CA United States

**Keywords:** HIV/AIDS, transgender women, SMS text messaging, peer health navigation, HIV Care Continuum

## Abstract

**Background:**

Many transgender women with HIV achieve suboptimal advancement through the HIV Care Continuum, including poor HIV health care usage, retention in HIV medical care, and rates of viral suppression. These issues are exacerbated by comorbid conditions, such as substance use disorder, which is also associated with reduced quality of life, increased overdose deaths, usage of high-cost health care services, engagement in a street economy, and cycles of incarceration. Thus, it is critical that efforts to End the HIV Epidemic include effective interventions to link and retain transgender women in HIV care through full viral suppression.

**Objective:**

This study builds on the promising findings from our two Health Resources and Services Administration-funded demonstration projects, The Alexis Project and Text Me, Girl!, which used peer health navigation (PHN) and SMS text messaging, respectively, for advancing transgender women with HIV to full viral suppression. Though the effectiveness of both interventions has been established, their comparative effectiveness, required resources or costs, cost-effectiveness, and heterogeneous effects on subgroups, including those with substance use disorder, have not been evaluated. Given the many negative personal and public health consequences of untreated or undertreated HIV, and that HIV services for transgender women are frequently delivered in resource-limited, community-based settings, a comprehensive economic evaluation is critical to inform decisions of stakeholders, such as providers, insurers, and policy makers.

**Methods:**

Text Me, Alexis! is a 3-arm randomized controlled trial. Participants (N=195) will be randomized (1:1:1) into: PHN alone (n=65), SMS text messaging alone (n=65), or PHN+SMS text messaging (n=65). Using the same time points as the Health Resources and Services Administration demonstration projects, the repeated-measures design will assess participants at baseline, 3, 6, 12, and 18 months post randomization. Over the course of the 90 days, participants in the PHN arm will receive unlimited navigation sessions; participants in the SMS text messaging arm will receive 270 theory-based SMS text messages (3 messages daily) that are targeted, tailored, and personalized specifically for transgender women with HIV; and participants in the PHN+SMS text messaging arm will receive a combined PHN and SMS text message intervention. The desired outcome of Text Me, Alexis! is viral suppression and cost-effectiveness.

**Results:**

Recruitment began on April 10, 2024, and the first participant was enrolled on April 11, 2024. Data collection is expected to be completed in July 2027. Primary outcome analyses will begin immediately following the conclusion of the follow-up evaluations.

**Conclusions:**

Transgender women are a high-priority population for reaching End the HIV Epidemic goals. Findings have the potential to improve individual and population health outcomes by generating significant improvements in viral suppression among transgender women and guiding service provision and public policy.

**International Registered Report Identifier (IRRID):**

DERR1-10.2196/65313

## Introduction

### Background

Current national data suggests that over 14% of transgender women in the United States are currently living with HIV [1], a rate at least 30 times higher than that observed in the general population aged 13 years and older (0.44%) [2]. National data on transgender women with HIV demonstrate lower rates of linkage to HIV care, retention in care, antiretroviral therapy (ART) uptake, ART adherence, and viral suppression than cisgender men and women [3,4]. Thus, any concerted effort to End the HIV Epidemic must include effective interventions to link and retain transgender women in HIV care through durable viral suppression [5,6]. The HIV prevalence rate of transgender women in Los Angeles County (LAC) exceeds national prevalence estimates, with an estimated 33% of transgender women with HIV, of whom only 58% achieved sustained viral suppression in 2022 [7]. As such, transgender women have been identified as a high-priority population, and LAC as a priority county for Ending the HIV Epidemic [6-8].

Transgender women in the United States experience numerous barriers to retention in HIV care, ART adherence, and viral suppression, including structural determinants of health, such as poverty and housing instability [9,10]; lack of access to health insurance [11]; transphobic stigma and discrimination from providers, including HIV specialists [11-13]; and disproportionate rates of individual-level health disparities including lack of perceived support [14], experiences of violence [15], cycles of incarceration [10,16], and untreated or undertreated substance use disorders (SUD) and mental health disorders, which are highly comorbid [17,18]. A 17-year comparison study in LAC demonstrated that these conditions have worsened among transgender women, with decreased levels of income and housing stability, increased incidents of physical harassment and abuse, and increased rates of HIV and sexually transmitted infections [19].

Evidence demonstrates that transgender women who are successfully linked and retained in HIV care go on to achieve rates of viral suppression similar to that of cisgender men and women, confirming linkage and retention to HIV care as critical intervention outcomes [20,21]. Among transgender women, access to gender-affirming resources and care [6,13], greater medication self-efficacy [22], and tailored HIV messaging (eg, addressing fears of ART or hormone drug-drug interactions) [23-25] are all associated with higher odds of ART uptake and adherence.

As an intervention modality, peer health navigation (PHN) is considered generally well-suited for application among transgender women, as it can be tailored to each participant’s needs, and is premised on increasing participants’ self-efficacy [20,26,27]. Interventions including PHN have been shown to be efficacious in improving rates of HIV care engagement, ART adherence, and viral suppression among transgender women [28-30]. However, due to the intensity of PHN, some transgender women may prefer a lower-intensity intervention such as SMS text messaging to deliver gender-affirming, transspecific SMS text messages, which are based on theories to improve self-efficacy and avoid or reduce health risks. SMS text messaging is a viable option since telehealth and technology-based interventions have demonstrated both acceptability and effectiveness among transgender women [23,28,29,31,32].

The promising findings gleaned from our team’s two Health Resources and Services Administration-funded Special Projects of National Significance demonstration projects guided the Text Me, Alexis! randomized controlled trial design. Reback et al [33] demonstrated that increased attendance to PHN sessions was associated with significant and sustained (ie, through 18 months) achievement of both behavioral (coefficient range 0.12-0.38) and biomedical (coefficient=0.10) HIV milestones (all *P*≤.01); 85% were linked to HIV care, and 83% of the participants that enrolled detectable and achieved a 1 log viral load reduction went on to achieve viral suppression [28]. Additionally, Reback et al [24] produced significant and sustained (ie, through 18 months) overall increases in ART uptake, self-reported ART adherence as “excellent,” and achievement of an undetectable viral load defined as ˂200 copies/mL (49% vs 77%, 5% vs 38%, 35% vs 52%, all *P*≤.001) [25]. The Alexis Project has been included in the Substance Abuse and Mental Health Services Administration’s intervention guide for persons with substance use and mental health disorders, and Text Me, Girl! has been included in the Ryan White HIV/AIDS Best Practices Compilation. Thus, the appropriate next step was Text Me, Alexis!, the randomized controlled trial to simultaneously assess the relative efficacy of these interventions, and their respective costs and benefits to determine their efficiency and inform widescale implementation.

An economic evaluation was included to inform “real-world” resource allocation decisions faced by relevant stakeholders. Economic value (ie, the extent to which a stakeholder’s resources are efficiently allocated) is a fundamental concern for any intervention targeting transgender women with HIV, given the number of persons in need and the fact that services for transgender women are frequently delivered in resource-limited, community-based settings [3,19,20].

### Primary and Secondary Aims

The primary aim of the Text Me, Alexis! study is to determine the comparative effectiveness of PHN alone, SMS text messages alone, and PHN+SMS text messaging combined with the goal of viral suppression and cost-effectiveness, and to identify the resources (eg, time and materials) required to prepare for, implement, and sustain each intervention, and estimate the associated costs. Further, the study will conduct a comprehensive cost-effectiveness analysis to determine the relative value of each intervention from the health care–sector, state policy makers, and societal perspectives. The secondary aim of the study is to determine heterogeneous intervention effects of PHN alone, SMS text messaging alone, and PHN+SMS text messaging due to social and structural determinants of health (eg, poverty, housing insecurity, food scarcity, educational attainment, and lack of insurance) and differing individual-level characteristics (eg, racial or ethnic identity, age, SUD—by type, and time since HIV diagnosis) among transgender women with HIV.

## Methods

### Study Design

**Figure 1:**
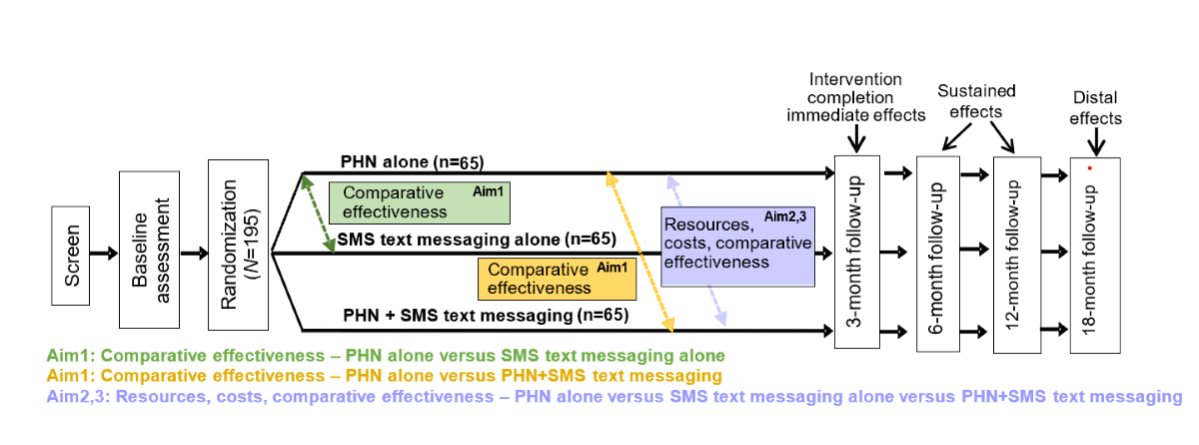
Comparative-effectiveness and cost-effectiveness design. PHN: peer health navigation.

Following screening, informed consent, and baseline assessment, participants (N=195) are randomized (1:1:1) into: PHN alone (n=65), SMS text messaging alone (n=65), or combined PHN+SMS text messaging (n=65). The 3-arm repeated-measures design will assess participants at baseline, 3 (immediate effects), 6, 12 (sustained effects), and 18 (distal effects) months post randomization to determine the relative effectiveness of the interventions, including heterogeneous treatment effects across subgroups and over time, the implementation and sustainment costs of each intervention, and their cost-effectiveness relative to one another. The study uses an “intent-to-treat” design whereby all assessments are administered to all participants regardless of their level of participation or retention in the study (Figure 1).

### Study Arms

#### PHN

**Figure 2:**
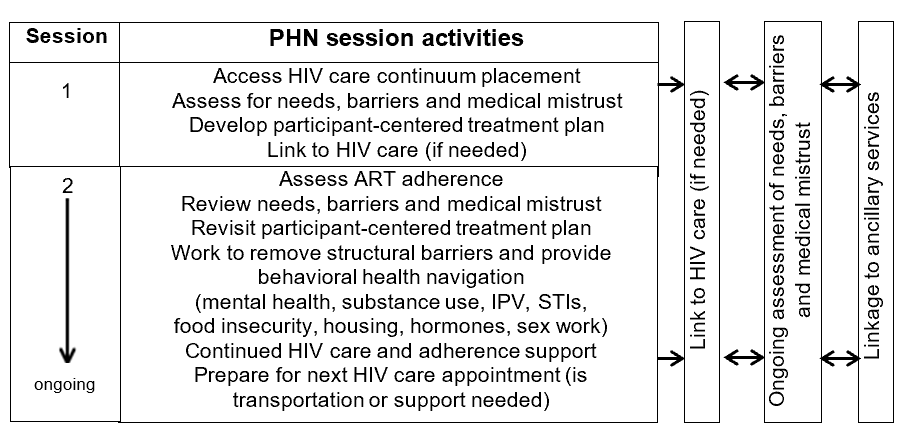
PHN intervention delivery system. ART: antiretroviral therapy; IPV: intimate partner violence; PHN: peer health navigation; STI: sexually transmitted infection.

PHN is based on the theoretical foundation of social cognitive theory (SCT). Participant-centered PHN helps to (1) identify barriers to HIV care, (2) identify and link participants to needed auxiliary services, and (3) increase participants’ self-efficacy in working with HIV care providers and other social service and treatment facilities. PHNs do not provide counseling or psychotherapy; they work with each participant to successfully navigate complicated health care and social service systems. The PHN intervention uses an individualized, participant-centered treatment plan with the goals of removing multiple and complex barriers that can impede linkage to and retention in HIV care and medication adherence to achieve virological suppression. Each participant works with a PHN to develop a participant-centered treatment plan and get linked to HIV care or other needed auxiliary physical, mental health, and psychosocial services (eg, hormone therapy, dental care, hepatitis testing or care, SUD treatment, mental health treatment, legal services, and job training or development; Figure 2). A priority of the first session is to schedule an HIV care appointment for the participant if needed. The PHN also works with each participant to establish HIV self-efficacy regarding her treatment plan. To establish an immediate connection, participants are introduced to a PHN immediately following randomization. PHN sessions are unlimited for 90 days.

#### SMS Text Messaging

The SMS text messages are based on, and equally distributed across, three theories: social support theory (SST) [34-36], social cognitive theory [37,38], and health belief model (HBM) [39]. Table 1 illustrates how the SMS text message library was developed to have a theoretical foundation and be transculturally responsive. Participants receive three daily, theory-based SMS text messages for 90 days (270 unique scripted messages); participants do not receive the same scripted SMS text message twice. SMS text messages are evenly arrayed across: (1) HIV Care Continuum (HIV positivity/physical and emotional health, linkage/retention in HIV Care, and ART adherence/viral load suppression); and (2) theoretical foundation (SST, HBM, or SCT; Table 2).

SMS text messages are transmitted through gradual automation administration daily, including weekends, in real time, within a 10-hour period, and every 5 hours. Optimum hours were determined to be noon, 5 PM, and 10 PM, though participants may personalize the schedule to any 10-hour period and can choose to have the messages delivered through their cell phone or email. Both dosing (ie, three messages per day) and timeframe (ie, 10 hours) were determined during our prior SMS text messaging studies [23,25]. The intervention was designed to be cost-efficient, sustainable, and easily scaled by health clinics or community agencies. The automated SMS text message delivery system was developed by Dimagi [40]. Participants are asked to notify a research assistant immediately if they lose their cell phone or change their phone number or email address.

**Table 1:** Sample Text Me, Girl! SMS text messages: displays theoretical foundation, HIV Care Continuum placement, and adaptation from a general message to a transspecific message.

Theoretical foundation	HIV Care Continuum	General message	Transspecific message
Social support theory	HIV positivity/physical and emotional health	Take care of yourself	Trans pride is taking care of yourself
Health belief model	Linkage/retention in HIV care	See your doctor	Protect your trans beautiful body, see your doctor
Social cognitive theory	ART^a^ medication adherence	Take your meds	Take your meds, girl! You can do it!

^a^ART: antiretroviral therapy.

**Table 2:** SMS text message content by theoretical foundation.

			HIV Care Continuum							Total
			HIV positivity/physical and emotional health		Linkage/retention in HIV care		ART^a^ adherence/viral load suppression			
**Theoretical foundation**										
	Social support theory	30		30		30		90		
	Health belief model	30		30		30		90		
	Social cognitive theory	30		30		30		90		
Total			90		90		90		270	

^a^ART: antiretroviral therapy.

#### PHN+SMS Text Messaging (Combined)

Participants in the PHN+SMS text messaging arm receive the same PHN and SMS text messaging interventions described above, but in concert to determine the effectiveness of the combined intervention when compared to PHN or SMS text messaging alone.

### Theoretical Mechanisms of Behavioral Change

The theoretical foundations of the interventions serve as mechanisms of behavior change, increasing advancement through the HIV Care Continuum and maximizing potential public health impacts.

#### SST

According to SST, social support encompasses instrumental, emotional, and informational assistance. These forms of social support have been shown to mediate the relationship between stressful events and health outcomes [34-36].

#### HBM

HBM asserts that believing specific health behaviors can reduce threats to health predicts one’s likelihood of engaging in protective health behaviors. The HBM is most effective when informative messages are culturally appropriate to the target population [39].

#### SCT

SCT posits interactive causal relationships among personal determinants, behavior, and environmental influences [37,38], and is designed to improve participant self-efficacy. Effective HIV care interventions must increase individuals’ self-efficacy and guide them in developing self-regulation skills, offer practice and feedback opportunities, and engage resources to maintain health-promoting behavior change.

### Participants

Inclusion criteria are (1) identifying as a transgender woman; (2) being 18 years or older; (3) having a verified HIV-positive serostatus; (3) not currently in HIV care, or had not had an HIV care visit in the previous 6 months, or had a viral load of ≥200 copies/mL on her last laboratory test result, or not currently prescribed ART, or prescribed ART but does not rate her ability to take all her medications as “excellent”; and (4) ability to receive daily SMS text messages on either a personal cell phone or via an email account. Potential participants must be able and willing to provide informed consent and comply with study requirements. Individuals are excluded if they do not meet all eligibility criteria.

### Recruitment and Enrollment

Six recruitment strategies are used to ensure a diversity of participants are enrolled. (1) Web-based: banner ads and digital flyers will be placed through geo-mapping on appropriate websites and social media platforms and are optimized for mobile platforms at 300×250 pixels. (2) Print media: local ads will be placed in print media for transgender women. (3) Outreach: 2 research assistants conduct outreach in identified areas where transgender women congregate. Optimal sites, days, and times have been identified, including bars or clubs, motels, parks, boulevards, street corners, mini markets, boutiques, wig shops, electrolysis offices, salons, and lingerie stores. Outreach locations are continually modified through ongoing community mapping and input from the Community Advisory Board. (4) Poster advertisement and club cards: posters are placed throughout the research site and community collaborating sites to inform potential participants how to receive further information. Club cards will be distributed at dance clubs, bars, and transspecific events. (5) In-reach: potential participants often drop into the research site to inquire about services or to receive a daily hot meal. (6) Long-chain referral: current study participants are asked to recruit a maximum of three potential new participants. All recruitment and promotional activities are discussed at Community Advisory Board meetings.

Potential participants who inquire about the study are scheduled for intake within 48 hours. At intake, potential participants are screened for eligibility, complete the informed consent process, and take an informed consent quiz to verify their understanding of study procedures. At baseline, potential participants complete the baseline assessment, collect biospecimens, and are randomized to an intervention arm. Potential participants are considered enrolled and given a study ID number following randomization.

### Randomization

Stratified block randomization with random block sizes is used to assign participants to each of the 3 study arms. To ensure balance with respect to certain covariates, participants are grouped by three stratification factors: (1) age (<35 and ≥35 years), (2) race or ethnicities (Latinx, all other race or ethnicities), and (3) HIV Care Continuum placement (linked, not linked to HIV care). Block randomization helps ensure balance in the number of participants assigned to each arm, while random block sizes make the sequence of assignments less predictable to research staff.

### Measures

All data will be collected on an audio computer-assisted self-interview administered via the Qualtrics system. The following describes the measures used to address the study’s specific aims.

#### Diagnostic and Statistical Manual of Mental Disorders (Fifth Edition)

The *DSM-5* (*Diagnostic and Statistical Manual of Mental Disorders* [Fifth Edition]) diagnostic items are necessary to make a determination of mild, moderate, or severe SUD. These findings are used to describe the sample characteristics and to determine the extent and effects of these individual-level health disparities as barriers to advancement along the HIV Care Continuum.

#### HIV Health Assessment

The assessment records demographics (eg, sexual identity, age, and race or ethnicity), educational attainment, housing status, access to insurance, HIV treatment status (including the position in the HIV Care Continuum), HIV medication status (including medication type and dose), and self-reported ART adherence.

#### The Los Angeles Transgender Health Survey

The instrument consists of seven modules: screening, sociodemographic characteristics, health care access and medical history, sexual behaviors (at all stages of gender transition), drug and alcohol use, legal and psychosocial issues, and HIV prevention.

#### Substance Use Frequency

This brief assessment assesses substance use, injection drug use, and injection protocols in the past 30 days.

#### HIV Treatment Adherence Self-Efficacy Scale

The HIV Treatment Adherence Self-Efficacy Scale (HIV-ASES) consists of 12 items assessing participants’ self-efficacy to adhere to their HIV medication regimen, to measure behavior change associated with SCT [41].

#### HIV Treatment Optimism Scale

The HIV Treatment Optimism Scale is a 19-item scale associated with components of the HBM (perceived susceptibility to disease, perceived severity of the disease, perceived benefits of preventive behavior, and barriers to preventive behavior) [42].

#### Inventory of Socially Supportive Behaviors

The Inventory of Socially Supportive Behaviors (ISSB) is a 40-item scale measuring instrumental, emotional, and informational dimensions of SST [36,43].

#### Rapid HIV Antibody Test

Potential participants are administered a rapid HIV antibody test (INSTI HIV 1/HIV 2) during the screening process to verify HIV-positive status. Participants who show documentation of HIV-positive serostatus (eg, laboratory results, ART prescription) are not given an HIV antibody test.

#### Urine Drug Screen

Urine samples are tested using a 5-panel urine dip card [44], with drug detection cut-off values at: amphetamines (1000 ng/mL), cocaine (300 ng/mL), opiates (300 ng/mL), methamphetamines (500 ng/mL), and tetrahydrocannabinol (50 ng/mL). Valid samples are indicated by the temperature of the sample (33 °C to 36 °C) [44].

#### Viral Load Test

Participants receive a viral load test at each time point to assess virologic suppression or control as indicated by an undetectable HIV-1 level on the Aptima HIV-1 Quant Dx assay, which is the lower limit of quantification of ≤30 copies/mL [45]. Participants who access their electronic health records and provide viral load results within 14 days of the assessment are not reassessed with viral load testing performed as part of the study.

#### Drug Abuse Treatment Cost Analysis Program

The Drug Abuse Treatment Cost Analysis Program is a standardized, customizable tool designed to help identify intervention resources across diverse settings for the purposes of estimating the implementation and sustainment costs associated with the intervention [46].

#### Nonstudy Medical and Other Services

The usage of health care services by participants is self-reported using a time-anchoring methodology via the Nonstudy Medical and Other Services form [47-49]. Health care services will include nonstudy: HIV care, inpatient, outpatient, and emergency department services; SUD treatment medications; residential and outpatient SUD treatment days; hospital SUD detoxification days; and mental health treatment visits. This information is measured for the 90 days prior to baseline, then “since the last assessment.” The use of nonmedical and other resources required for the economic evaluation from state policy maker and societal perspectives (eg, criminal-legal, labor productivity, and travel time to medical care) is also self-reported and collected via the Nonstudy Medical and Other Services form.

#### Patient-Reported Outcomes Measurement Information System-Preference

The PROMIS (Patient-Reported Outcomes Measurement Information System)-Preference (PROPr) measures a participant’s health-related quality-of-life across the following PROMIS domains: cognitive function abilities, depression, anxiety, fatigue, pain interference, pain intensity, physical function, sleep disturbance, and ability to participate in social roles and activities [50-52]. The PROPr is also capable of generating a health utility index value, based on the participant’s scores for each domain, that represents the general US population’s preference for the respondent’s current health state. PROPr has 5 levels for each domain, ranging from “no problems” to “extreme problems.” The health-utility value produced by PROPr can range from –0.022 to 1, where 0 represents death, 1 represents perfect health, and values below 0 represent states perceived to be worse than death. The health-utility value is used to calculate quality-adjusted life-years (QALYs) [50,53].

### Statistical Analyses

#### Aim 1

The primary outcome of HIV care will be viral suppression (defined as less than 200 copies/mL). Secondary outcomes include the HIV-ASES, the HIV Treatment Optimism Scale, the ISSB, and urine drug screen results (test results for each of the 5 substances identified in the urine drug screen will be treated as different indicators). These outcome variables will be assessed at baseline and 3, 6, 12, and 18 months post enrollment. The resulting dependent variables will fall into one of two categories: (1) dichotomous variables (primary outcome: virologic suppression, and secondary outcome: urine drug screen results), assumed to follow a binomial distribution; or (2) continuous random variables (secondary outcomes: HIV-ASES, HIV Treatment Optimism Scale, ISSB scores), assumed to follow a normal distribution. All distributional assumptions will be evaluated prior to the conduct of analyses, and statistical methods chosen accordingly. Each of these dependent variables will be separately regressed on treatment condition (PHN alone vs SMS text messaging alone vs PHN+SMS text messaging), a set of baseline covariates (individual-level demographic characteristics), and time-varying covariates (social and structural determinants of health), using hierarchical linear regression where responses at each time point are nested within individuals. All analyses will be conducted on available study-related data from all participants, regardless of whether or when they drop out of treatment. The effect of interest will be a time × treatment condition interaction effect which will estimate the differential course and impact of the three intervention modalities over the follow-up period.

A generalized linear mixed model (GLMM) [54-56] will be used to conduct analyses of all outcomes following an intent-to-treat approach. The GLMM is an ideal statistical procedure for analyzing a broad class of longitudinal outcomes, including costs. As described above, key indicators of HIV care will be regressed on treatment conditions and a series of baseline and time-varying covariates. Treatment condition will be used to predict the slope of time, creating a time × treatment interaction term that is the effect of interest in the proposed study. We will also report on the relationships between time-varying covariates and the outcome measures, including time-shifted analyses where the value of the covariate at a previous timepoint is used to predict the outcome measure at a subsequent timepoint. Hypothesis testing for any given outcome will involve fitting the statistical model of interest and testing the effect of interest, as well as all other estimable effects in the model. We will use an iterative model-building approach where the simplest model is fit to the data first, and additional explanatory factors are added iteratively, in order of theoretical importance. Likelihood ratio tests are used to determine if the more complicated of the 2 models is a significant improvement over the simpler model. Variables of interest such as those specified in Aim 1 will always be included in the final model as the statistical tests of those parameter estimates (be they significant or nonsignificant) are of primary interest to this study.

#### Aims 2 and 3

The economic analyses will follow well-established guidelines [57-59]. The study will incorporate all resources or costs associated with the PHN, SMS text messaging, and PHN+SMS text messaging interventions from the health care sector, state policy maker, and societal perspectives [58-60]. The health care sector perspective includes all formal medical costs incurred by the system on behalf of participants, including the cost of the intervention, and participant out-of-pocket costs. The state policy maker perspective is crucial to informing resource allocation decisions on behalf of the public, who is primarily responsible for funding health care among this underserved population, given data indicating that most transgender women are either public health care insurance beneficiaries or uninsured [11,19]; moreover, the direct costs associated with criminal-legal resources, social safety-net programs, etc are paid for using public funds. In addition to the resources or costs included in the state policy maker perspective, the societal perspective accounts for those associated with untreated or undertreated HIV and comorbid conditions, such as premature mortality, reduced labor productivity, and those incurred by victims of crime [61,62].

The resources required to implement and sustain each intervention in a “real-world” setting (Aim 2) will be estimated using a detailed microcosting analysis, guided by a tailored version of the Drug Abuse Treatment Cost Analysis Program. The microcosting analysis will consist of gathering relevant administrative data and conducting semistructured interviews with site personnel in order to capture quantitative data regarding the resources (time and materials) used to deliver the interventions. The intervention implementation phase is considered to be the time period from conception (including planning activities) until the “steady state.” Resources will be categorized as “fixed start-up” (incurred once), “time-dependent” (recurring, but does not vary with the number of participants), and “variable” (used every time a participant is served). The site visits and initial interviews will be conducted early in the study (~6 months following the first randomization). Follow-up interviews will be conducted virtually upon the study reaching a “steady state” (~12 months following the initial interview), to identify the time-dependent and variable resources required to sustain the intervention. “Steady state” will be determined with the assistance of site personnel. Implementation and sustainment costs will be estimated by assigning nationally representative price weights to the identified resources [58]. Research-specific costs will be excluded.

After estimating the implementation and sustainment costs associated with each intervention (PHN, SMS text messaging, and PHN+SMS text messaging), the relative value of each will be estimated according to the stakeholder perspective. This process entails capturing all relevant resources used by participants in each arm, assigning nationally representative price weights to them, estimating the predicted mean costs, and testing for differences between resource categories. Estimating the incremental costs between arms according to resource category allows for a careful evaluation of the downstream savings resulting from improvements in HIV care and reductions in related risk behaviors. These include savings resulting from reduced usage of high-cost health care (eg, emergency department visits and inpatient stays) and criminal-legal resources, as well as increased labor and other forms of productivity. Price weights will be derived from sources reflecting national “real-world” costs faced by state policy makers and society.

The primary outcome of the cost-effectiveness analysis (Aim 3) will be the incremental cost-effectiveness ratio (ICER), which will be calculated as the incremental, predicted-mean cost of a given intervention relative to an alternative, divided by the incremental predicted-mean effectiveness of the 2 interventions. The primary measure of effectiveness for the economic evaluation will be QALYs. The secondary measure of effectiveness will be advancement along the HIV Care Continuum. The QALY is a measure that combines the health-related quality-of-life associated with an individual’s health state and the time spent in that state and is recommended as the primary effectiveness measure in economic evaluation studies due to its ability to be compared across interventions and disorders [58,63]. In addition, generally accepted thresholds for defining value have been established for QALYs, unlike clinical measures [64,65]. The HIV Care Continuum is an important and widely accepted model or tool for assessing HIV care outcomes at both an individual and a public health level; thus, the additional cost required to achieve a one-step increase along the HIV Care Continuum for the average transgender woman with HIV will be a critically important clinical and policy-relevant measure. Two ICERs (one for each effectiveness measure) will be calculated for each stakeholder perspective at both 3 months (intervention completion; immediate effects) and 18 months (distal effects).

To help address censored data, we will model the person period and estimate all regressions using a multivariable GLMM. Separate regressions will be estimated to predict the mean value for each resource category, at each time period, by study arm. The statistical method of recycled predictions will be used to obtain the final predicted mean values [58]. Similarly, individual regressions will be used to predict the health utility index value and HIV Care Continuum steps gained for each participant, at each time point. QALYs gained will be estimated using the predicted health utility values and the area under the curve methodology [58]. The most appropriate distributional and link functions for each GLMM regression will be chosen according to the fit of the observed data [58].

To account for sampling uncertainty in point estimates, the *P* values and SEs will be estimated using nonparametric bootstrapping techniques within the multivariable framework combined with methods to address missing data based on recommended approaches [66]. All monetary values will be adjusted for inflation, and all measurements obtained beyond 12 months of baseline will be discounted for time preference using the recommended rate of 3% [58,65].

The most cost-effective strategy for each outcome measure (QALYs; HIV Care Continuum placement) will be determined using the rules of strong and extended dominance. Parametric methods based on parameters obtained from bootstrapping will be used to estimate cost-effectiveness acceptability curves for each ICER, which illustrate the probability that an intervention is cost-effective for different value thresholds [57].

#### Power Analysis

A simulation approach was used to conduct the power analysis and determine that N=195 (65 per arm) was an appropriate sample size to detect the effect of each intervention on our primary outcome, and the likelihood of virologic suppression over time [67]. Power by simulation is more flexible than traditional approaches because it can estimate power under a wide variety of circumstances, including when these assumptions are not met.

### Ethical Considerations

All study procedures are approved by the Western Institutional Review Board (Study #1352118; Institutional Review Board Tracking #20231531). This trial has been registered at ClinicalTrials.gov under the number NCT06408350. All procedures performed in studies involving human participants were in accordance with the ethical standards of the institutional or national research committee and with the 1964 Helsinki Declaration and its later amendments or comparable ethical standards. Informed consent was obtained from all individual participants included in the study.

## Results

Recruitment began on April 10, 2024, and the first participant was enrolled on April 11, 2024. Recruitment spans approximately 33 months; enrollment goals are approximately 6 enrolled participants per month. Data collection, including all follow-up assessments, is expected to be completed in July 2027.

## Discussion

### Principal Findings

Many transgender women have suboptimal advancement through the HIV Care Continuum, including poor HIV health care usage, retention in HIV medical care, and rates of viral suppression; moreover, these issues are exacerbated by comorbid conditions such as SUDs. The Text Me, Alexis! study is a comparative-effectiveness research trial with a comprehensive economic evaluation that builds upon the promising findings of two Health Resources and Services Administration-funded demonstration projects in order to identify the optimal intervention for advancing transgender women with HIV to full viral suppression. Though the effectiveness of the demonstration projects has been established, their comparative effectiveness, required resources or costs, cost-effectiveness, and heterogeneous effects on subgroups, including those with SUDs, have not been evaluated.

### Challenges

There are several challenges to the Text Me, Alexis! study. First, housing instability, substance use, engagement in sex work, and other individual-level, social, and structural disparities may interfere with study participation. The PHN alone and PHN+SMS text messaging arms were designed to address these issues and work with each participant to assess and minimize or remove barriers and link participants to an array of ancillary social services. Additionally, in the demonstration project, despite experiencing several health disparities including low educational attainment, low income, and housing instability, SMS text messaging alone demonstrated significant improvements in ART uptake, ART adherence, and achievement of an undetectable viral load, which were durable through 18-month follow-up. Second, episodes of short-term incarceration may interrupt study progress and follow-up assessment rates, due to factors such as actual or perceived participation in the street economy, or minor homeless infractions. Study staff monitor the public records database for participants who miss appointments. When an incarcerated participant is found, we begin a correspondence with the participant immediately upon release. Third, loss of ART or selling ART due to lifestyle needs (“diversion”)—ART adherence will be stressed through the participant-centered treatment plan and via adherence-specific SMS text messages, including strategies for keeping medication safe, and a discussion on how ART adherence outweighs selling the drug. Finally, fear of drug-drug interaction—some participants may be concerned about ART and gender-affirming hormone therapy interactions and prioritize gender-affirming hormone therapy over ART uptake or adherence. These concerns will be acknowledged and corrected in PHN sessions and SMS text messages.

### Conclusions

The public health significance of Text Me, Alexis! has the potential to be quite profound, as comparative- and cost-effectiveness research trials are critical steps in the development and adoption of scalable and effective HIV care intervention, especially among key populations that rely on service provision in resource-limited, community-based settings.

## Appendix

Multimedia Appendix 1 Summary Statement Peer Review Comments.

## Abbreviations

ART: antiretroviral therapy
DSM-5: Diagnostic and Statistical Manual of Mental Disorders (Fifth Edition)
GLMM: generalized linear mixed model
HBM: health belief model
HIV-ASES: HIV Treatment Adherence Self-Efficacy Scale
ICER: incremental cost-effectiveness ratio
ISSB: Inventory of Socially Supportive Behaviors
LAC: Los Angeles County
PHN: peer health navigation
PROMIS: Patient-Related Outcome Measurement Information System
PROPr: PROMIS-preference score
QALY: quality-adjusted life-year
SCT: social cognitive theory
SST: social support theory
SUD: substance use disorder

## Data Availability

The datasets generated during or analyzed during this study are available from the corresponding author upon reasonable request.
