# Supplementary material for: Comparative- and Cost-Effectiveness Research Determining the Optimal Intervention for Advancing Transgender Women With HIV to Full Viral Suppression (Text Me, Alexis!): Protocol for a Randomized Controlled Trial
Source: JMIR Res Protoc. 2025 Jan 23;14:e65313. doi: 10.2196/65313 (PMC11803334; doi:10.2196/65313)
Supplement: Multimedia Appendix 1 [file resprot_v14i1e65313_app1.pdf]

SUMMARY STATEMENT

(Privileged Communication)

PROGRAM CONTACT:
MINNJUAN Flournoy Floyd
301-827-6474
minnjuan.flournoyfloyd@nih.go
v

Release Date: 12/16/2021
Revised Date:

Application Number: 1 R01 DA056287-01

Principal Investigators (Listed Alphabetically):

MURPHY, SEAN M.
REBACK, CATHY J (Contact)

Applicant Organization: FRIENDS RESEARCH INSTITUTE, INC.

Review Group: PPAH
Population and Public Health Approaches to HIV/AIDS Study Section
AIDS - EXP. REV.

Meeting Date: 11/15/2021
Council: JAN 2022
Requested Start: 04/01/2022

RFA/PA: PA20-183
PCC: CM/MWF

Project Title: Comparative- and cost-effectiveness research determining the optimal intervention for advancing transgender women living with HIV to full viral suppression
SRG Action: Impact Score:34 Percentile:28
Next Steps: Visit https://grants.nih.gov/grants/next_steps.htm
Human Subjects: 30-Human subjects involved - Certified, no SRG concerns
Animal Subjects: 10-No live vertebrate animals involved for competing appl.
Gender: 2A-Only women, scientifically acceptable
Minority: 1A-Minorities and non-minorities, scientifically acceptable
Age: 3A-No children included, scientifically acceptable

Project Year	Direct Costs Requested	Estimated Total Cost
1	499,961	673,014
2	732,799	986,445
3	763,137	1,027,284
4	693,302	933,277
5	499,984	673,045
TOTAL	3,189,183	4,293,064

ADMINISTRATIVE BUDGET NOTE: The budget shown is the requested budget and has not been adjusted to reflect any recommendations made by reviewers. If an award is planned, the costs will be calculated by Institute grants management staff based on the recommendations outlined below in the COMMITTEE BUDGET RECOMMENDATIONS section.

1R01DA056287-01 Reback, Cathy

RESUME AND SUMMARY OF DISCUSSION: The applicant seeks to compare the effectiveness of Two projects in increasing viral suppression (VS) among transgender women (TW): a peer health navigation (PHN) intervention labeled Alexis Project and an SMS based intervention called Text Me, Girl. The premise for this application is strong; TW, especially those with co-morbid conditions and substance use disorder, bear an inordinate burden of HIV infections and are less likely to uptake ART and to persist in adherence. Though these two interventions were found effective when used alone; their comparative effectiveness, resources required, and cost effectiveness have not been established. The application is particularly innovative in assessing the cost impact and quality of life (QOL) changes in this population. It is further undergirded by some preliminary work with similar populations; the PI is excellent with a wealth of experience in research with TW population. She will be assisted by a cast of co-investigators with multidisciplinary and complementary skills; together, they form a very strong team. This MPI application is well justified; the PIs have clearly stated their roles and responsibilities and have set up appropriate protocols for communication and conflict resolution. The methods and analyses proposed are generally strong and well considered. There were, however, some minor to moderate concerns that moderately dampened enthusiasm for the application; these included the following: the prior research that supports this application did not have standard of care control groups, making it difficult to interpret results; furthermore, they had a contingency management component not present in the current design, potentially decreasing the effectiveness of the combination intervention; there is a lack of detail on how cost-effectiveness will be conducted; there is insufficient discussion of potential problems given dependencies of Aim 3 on effectiveness data in Aim 2; there were also differences of opinion regarding the justification for combining PHN+SMS testing since it is not supported by any preliminary data. Moreover, the sample size in each arm seems relatively small and the power calculation does not account for multiple hypothesis testing. Despite these concerns, the majority of the committee felt that this application could substantially advance the HIV care of TW by addressing VS; as such these reviewers assessed the application's potential impact as high to very high. The remainder assessed it as moderate to moderately strong.

DESCRIPTION (provided by applicant): Trans women (TW) achieve suboptimal advancement through the HIV Care Continuum,1-7 including poor HIV healthcare utilization,8,9 retention in HIV medical care,10-12 and rates of viral suppression.3,4,6,7,13,14 These issues are exacerbated by comorbid conditions, such as substance use disorder (SUD),15-22 which is also associated with reduced quality-of-life, and increased overdose deaths, utilization of high-cost healthcare services, engagement in a street economy, and cycles of incarceration.23,24 Drug use among TW has been demonstrated to be a barrier to HIV care and advancement along the HIV Care Continuum.16 Thus, it is critical that efforts to End the HIV Epidemic (EHE) include effective interventions to link and retain TW in HIV care through full viral suppression.25-27 This study builds on the promising findings from our two HRSA-funded demonstration projects, The Alexis Project28 and Text Me, Girl!,29 which utilized Peer Health Navigation (PHN) and SMS (i.e., text messaging), respectively, for advancing TW living with HIV to full viral suppression. Though the effectiveness of both interventions has been established, their comparative-effectiveness, required resources/costs, cost-effectiveness, and heterogeneous effects on subgroups, including those with SUD, have not been evaluated. Given the many negative personal- and public-health consequences of untreated/undertreated HIV, and that HIV services for TW are frequently delivered in resource-limited, community-based settings,30-33 a comprehensive economic evaluation is critical to inform decisions of stakeholders, such as providers, insurers, and policymakers. The "intent-to-treat" RCT will randomize participants (N=225) into: PHN alone (n=75), SMS alone (n=75), or PHN+SMS (n=75). Using the same time points as the HRSA projects, the repeated-measures design will assess participants at baseline, 3-, 6-, 12-, and 18-months post-randomization. The specific aims are: 1) Conduct a comparative effectiveness research trial to

determine the relative effectiveness of PHN vs. SMS vs. PHN+SMS in terms of: Primary (a) virologic suppression; and Secondary outcomes (b) HIV Treatment Adherence Self-Efficacy Scale scores; (c) the AIDS Health Belief Scale scores; (d) the Inventory of Socially Supportive Behaviors scores; and (e) urine drug screen results; 2) Identify the resources required to prepare for, implement, and sustain each intervention, and estimate the associated costs; 3) Conduct a comprehensive cost-effectiveness analysis to determine the relative value of each intervention from the healthcare-sector, state-policymaker, and societal perspectives; and, a Secondary Aim to determine heterogeneous intervention effects across interventions due to social and structural determinants of health and individual-level characteristics. TW are a high-priority population for reaching EHE25-27 goals and Los Angeles County (the study location) is an EHE priority County.34,35 Findings have the potential to improve individual and population health outcomes by generating significant improvements in viral suppression among TW and guiding service provision and public policy.

PUBLIC HEALTH RELEVANCE: Trans women (TW) achieve suboptimal advancement through the HIV Care Continuum, including poor HIV healthcare utilization, retention in HIV medical care, and rates of viral suppression and these issues are exacerbated by comorbid conditions such as substance use disorder. This application will conduct a comparative-effectiveness research trial with a comprehensive economic evaluation of two HRSA-funded demonstration projects, to identify the optimal intervention for improving rates of viral suppression among TW living with HIV. The public health significance is profound, as comparative- and cost-effectiveness research trials are critical steps in the development and adoption of scalable and effective HIV care intervention, especially among key populations that rely on service provision in community-based settings.

CRITIQUE 1

Significance: 2
Investigator(s): 2
Innovation: 3
Approach: 7
Environment: 1

Overall Impact: This R01 project aims to conduct a comparative-effectiveness research trial with an economic evaluation of two HRSA-funded demonstration projects, to identify the optimal intervention for improving rates of viral suppression among trans women (TW) living with HIV. This proposal addresses a very significant issue in the field of HIV care for TW, yet the overall impact is only moderate because there are several weaknesses noted, and the rigor of the prior research is not strong. The prior research papers did not have standard of care control groups, and they had a component (contingency management, CM) which has been left out of the current design. Thus, the current design is in clear contradiction with the PI's previous findings. The project is innovative with respect to using comparative effectiveness with HRSA-funded demonstration projects' results. However, the project is not technically innovative as it uses standard methods for comparative- and cost-effectiveness research. The approach has some strong aspects such block randomization and prior trials with similar populations. However, there were several weaknesses related to design (untested combination intervention without CM), power calculation (relying on simulations and not accounting for multiple hypothesis), not enough detail for preventing bias in CEA societal approach, and lack of discussion of potential problems (if effectiveness is null). The weaknesses related to the main design are most concerning. The problem with this is that they make an assumption that a combined intervention (peer health navigators plus SMS texting will have effects), and that does not seem to be supported by adequate data. The investigators are well-trained in sociology, economics, medicine; and the facilities and environment are

appropriate. In sum, despite the high significance, there are several weaknesses in the approach that dampen our enthusiasm for the project, and the potential overall impact is deemed moderate at best.

1. Significance:

Strengths

- Trans women (TW) have disproportionate burden of HIV infection and achieve suboptimal outcomes through the HIV Care Continuum including poor HIV healthcare utilization, low retention in HIV medical care, and reduced rates of viral suppression.
- A comparative-effectiveness and cost-effectiveness analysis (CEA) approach has the potential to maximize efficiency at the societal level.

Weaknesses

- No mention of the approximate total population of TW in the US, or the total burden of disease in the target population.

2. Investigator(s):

Strengths

- MPI, Reback, is trained in sociology and has relevant previous projects including NIH R01 level funding and HRSA funding.
- MPI, Murphy, has training in economics.
- The other team members have training and experience in medicine, HIV care, psychology, technology-based interventions.
- The MPI plan is well justified and the roles of the MPIs are well delineated; there is a protocol developed, as well as previous collaboration, to ensure good communication. There is a contingency plan to address any conflicts that may arise.

Weaknesses

- MPI Murphy seems to be extremely busy, listing 21 currently funded projects as PI or co-I. (moderate).

3. Innovation:

Strengths

- Design combines and extends two prior HRSA demonstration projects.
- Emphasis on comparative- and cost-effectiveness evaluation is innovative.

Weaknesses

- Design drops an important component of the demonstration project: contingency management.
- Using QALYs for a CEA is not new.

4. Approach:

Strengths

- An intent-to-treat RCT will randomize participants (N=225) into peer health navigators (PHN) alone (n=75), SMS alone (n=75), or PHN+SMS (n=75).

- Primary outcome is virologic suppression.
- Previous HRSA-funded demonstration project results have been published.
- Comparative effectiveness trial has stratified block randomization with random block sizes used to assign participants to each of the three study arms.

Weaknesses

- The Text Me Girl evaluation did not have a standard of care control arm. (moderate)
- The Alexis evaluation was non-random and did not have a control group; also, the intervention combined PHN and contingency management, so it is not clear what component or active ingredient would be responsible for any observed changes. (moderate)
- Removing the CM component is a political decision, clearly not based on the PI's own research findings. If CM was a biomedical intervention, given the overwhelming positive evidence, it would be unethical to deny this treatment to the populations in need. (major)
- Adding the PHN + SMS without the CM is not justified scientifically. The texting intervention also used incentives (for up to $380) and it did not have a SoC control arm. Thus, it could be that the incentives were responsible for the effects, not the SMS. (major)
- Not enough detail to ensure that incorporating the societal perspective does not suffer from publication bias and other biases when accounting for costs associated with untreated or undertreated HIV and comorbid conditions, such as premature mortality, reduced labor productivity, and costs incurred by victims of crime. (moderate)
- Power analysis relies on modeling methods because data for main effects is not available in the form needed for the proposed research. Also, the power estimates do not account for multiple hypothesis testing of effect interactions (with several potential effect modifiers: poverty, housing insecurity, food scarcity, educational attainment, lack of insurance, etc.) (moderate)
- Aim 3 on cost effectiveness is dependent on finding a positive difference in comparative effectiveness in aim 2. However, since all arms have a potentially active ingredient that may work for all (i.e., participants' incentives), then there may be no difference in the comparative effectiveness. Thus, the cost effectiveness would not be identified. (moderate)

5. Environment:

Strengths

- Friends Research Institute and the Weill Cornell Medical College provide excellent environment for the proposed research.

Weaknesses

- None noted.

Study Timeline:

Strengths

- Sufficiently detailed and appropriate.
- The timeline is appropriate regarding the start-up activities (6-9 months), the anticipated rate of enrollment (about 7 participants/month), the planned follow-up assessment (until month 57).

Weaknesses

- The projected timeline may not be fully feasible and sufficiently well justified for the final data analysis and the cost effectiveness analysis for which there will be only 3 months (since the last follow up visit is in month 57 out of a total of 60 months project period).

Protections for Human Subjects:

Acceptable Risks and/or Adequate Protections

- Appropriate.

Data and Safety Monitoring Plan (Applicable for Clinical Trials Only):

Acceptable

- Appropriate.

Inclusion Plans:

- Sex/Gender: Distribution justified scientifically
- Race/Ethnicity: Distribution justified scientifically
- For NIH-Defined Phase III trials, Plans for valid design and analysis: Not applicable
- Inclusion/Exclusion Based on Age: Distribution justified scientifically
- Appropriate.

Vertebrate Animals:

Not Applicable (No Vertebrate Animals)

Biohazards:

Not Applicable (No Biohazards)

Resource Sharing Plans:

Acceptable

Authentication of Key Biological and/or Chemical Resources:

Acceptable

Budget and Period of Support:

Recommend as Requested

CRITIQUE 2

Significance: 1
Investigator(s): 1
Innovation: 2
Approach: 3

Environment: 1

Overall Impact: This is a well-conceptualized and presented R01 application by a team of experienced investigators to conduct a comparative-effectiveness research (CER) trial, with an economic evaluation component, to identify the optimal intervention for improving viral suppression among transgender women (TW) living with HIV. The study will be a RCT that assesses comparative-effectiveness, implementation and sustainment resources/costs, cost-effectiveness of two established interventions, alone and in combination. The two interventions are HRSA projects, Text Me, Girl! and The Alexis Project, both of which were led by PI Reback. Participants (N=225) will be randomized 1:1:1 into PHN alone (n=75), the Alexis Project, SMS alone (n=75) the Text Me Girl! project, or both PHN+SMS (n=75) both projects. Modeled on the HRSA demonstration projects, the three-arm, repeated-measures design will assess participants at baseline, 3- (immediate effects), 6-, 12- (sustained effects), and 18- (distal effects) months post-randomization to assess the comparative- and cost-effectiveness of the interventions, as well as relative intervention effects across subgroups, and over time. The study team is excellent, and the outcomes include health-related quality of life (HRQoL), which is an important stand-alone outcome worthy of study for TW and all people. The main CER RCT outcomes are viral suppression; secondary outcomes include the HIV Treatment Adherence Self- Efficacy Scale (HIV-ASES), the AIDS Health Belief Scale (AHBS), the Inventory of Socially Supportive Behaviors (ISSB), and urine drug test results for 5 substances). The economic analysis outcomes include resources/costs required to implement and sustain each intervention, as well as the value of each (PHN, SMS, PHN+SMS) relative to the others. They will extrapolate the "downstream savings" from improvements in HIV care and reductions in outcomes such as utilization of high-cost healthcare and criminal-legal resources, increased labor productivity, etc. The analytic plan presents approaches to missing data and potential challenges. Altogether the project promises to deliver important needed information for supporting a population subgroup that is marginalized and highly adversely affected by HIV. The study, led by a highly capable study team, has potential for high impact with minor adjustments to the approach.

1. Significance:

Strengths

- The need for information on the comparative effectiveness of interventions to prevent HIV transmission and optimize health and well-being among TW living with HIV cannot be overstated.
- The investigators make an excellent case for the need for the research epidemiologically and econometrically.
- The study interventions also take substance use, and other structurally conditioned health behaviors, into account – a critical component of the interventions.
- The scientific premise is very strong, particularly the role of peer health navigation (PHN) which the Alexis Project integrates.
- There is a strong need for economic evaluations as evidence of cost-effectiveness is critical during (supposed or societally-chosen) resource restrictions.

Weaknesses

- None noted.

2. Investigator(s):

Strengths

- The team is excellent and well-positioned to conduct the research. Dr. Reback (contact MPI) is trained in sociology and has a track record of NIH funding and managing large R01 grants. They also have a strong track record working with transgender women, an expertise and effort-built capital/trust, without which the study will not be successful.
- Dr. Murphy (MPI) is an Associate Professor of Population Health Sciences at Weill Cornell Medical College; Co-Director of the CHERISH Methodology Core (Center for Health Economics of Treatment Interventions for Substance Use Disorder, HCV, and HIV; and a health economist/econometrician.
- The investigator designed the interventions to be assessed in the CER study and thus are well-positioned to conduct the research proposed.

Weaknesses

- None noted.

3. Innovation:

Strengths

- Independently none of the components are particularly innovative, but together and combined with the cost effectiveness approach, the study is innovative and will add needed information.

Weaknesses

- None noted.

4. Approach:

Strengths

- The CER RCT will use a 3-arm repeated measures (3/6/12/18 months follow-up) design with an intent to treat analytic plan.
- They plan to assess heterogeneous treatment effects across subgroups and time.
- The CER component is well-presented.
- Each intervention is theoretically grounded and supported by prior empirical evaluation.
- The study is conducted in partnership with an established clinic and there is a CAB to advise the research team.
- The team has a track record of successful recruitment via a range of methods (inreach, long chain referrals, etc.) and are trusted.

Weaknesses

- The number of participants per arm is quite small, although the underlying population is not that large. The power analysis suggests that this is powered properly and the team has a track record of recruiting and retaining women in study so this is not a big concern.
- The interventions are theoretically grounded although the intervention mechanisms are not particularly well-described in this proposal, although the supporting research illustrates them in more detail.

- While stigma and discrimination are assessed it is unclear how more modern manifestations are addressed in the interventions and measurement battery. The measures are based on older assessments and have been updated, so it seems that they have been adapted as well.

5. Environment:

Strengths

- The environment is optimal for conducting the study.

Weaknesses

- None noted.

Study Timeline:

Strengths

- The timeline is well-described and adequate.

Weaknesses

- None noted.

Protections for Human Subjects:

Acceptable Risks and/or Adequate Protections

Data and Safety Monitoring Plan (Applicable for Clinical Trials Only):

Acceptable

- The DSMP/B is acceptable.

Inclusion Plans:

- Sex/Gender: Distribution justified scientifically
- Race/Ethnicity: Distribution justified scientifically
- For NIH-Defined Phase III trials, Plans for valid design and analysis: Not Applicable
- Inclusion/Exclusion Based on Age: Distribution justified scientifically
- All aspects of inclusion are acceptable.

Vertebrate Animals:

Not Applicable (No Vertebrate Animals)

Biohazards:

Not Applicable (No Biohazards)

Resource Sharing Plans:

Acceptable

Budget and Period of Support:

Recommend as Requested

CRITIQUE 3

Significance: 3
Investigator(s): 1
Innovation: 3
Approach: 2
Environment: 1

Overall Impact: The proposal "Comparative- and cost-effectiveness research determining the optimal intervention for advancing transgender women living with HIV to full viral suppression" is a well-integrated sequence of a small clinical trial, a resource analysis, and a cost-effectiveness analysis, with a possible additional aim of assessing heterogenous treatment effects. The population focus of the proposal is trans women with HIV in Los Angeles, and the interventions are PHN and SMS. The proposal is very well conceived and put together.

1. Significance:

Strengths

- Trans women (TW) have very high prevalence of HIV (16-27%) per the investigators - hard to fully believe).
- TW have low rates of viral suppression.
- Getting a comprehensive analysis of approaches to getting TW engaged in care is important.

Weaknesses

- Similar to many studies, it is unclear how big is the population addressed. To take an extreme example, if there were only 10 TW in LAC, and 3 of them had HIV, that would be a very high prevalence, but a very small group. That size makes a difference for the significance.

2. Investigator(s):

Strengths

- Excellent expertise and experience in community-based interventions and CEA of interventions for SUD.
- MPI plan is justified and detailed.

Weaknesses

- While a trial of 225 folks is manageable, it seems the team for supporting this trial is pretty skeletal. More would be useful.

3. Innovation:

Strengths

- Getting CEA done directly with a trial is, unfortunately, rare, and having this designed and baked into the trial up front is excellent.

Weaknesses

- Interventions are, effectively, the same ones previously used in the demonstration studies.

4. Approach:

Strengths

- Plans for recruitment, randomization, and ITT analysis of CER portion of study are excellent.

Weaknesses

- The amount of information collected at each visit seems burdensome, and in addition many of the measures only remotely address the primary outcomes of interest. Trimming that down to the most essential measures would improve data quality without compromising on the study results.
- When collecting data for societal perspective of a CEA, all relevant costs and benefits should be taken into account. In a population like this with high rates of disconnection from the formal health care system, there are hints of costs that are very salient to the TW but are not collected in this study (in part because they are hard to fully capture). Things like distrust of the medical system; social norms that do not favor receiving health care; a lack of desire to work for better health. Some of these would be important to capture.

5. Environment:

Strengths

- FCC is an excellent partner for this project.
- Weill has an excellent center for economic evaluations.

Weaknesses

- None.

Study Timeline:

Strengths Strengths

- Excellent plans for recruitment make this feasible in the time frame. Start-up activities are allocated adequate time, recruitment seems feasible. The projected timeline is feasible and well justified.

Weaknesses

- None noted by reviewer.

Protections for Human Subjects:

Acceptable Risks and/or Adequate Protections

- Relaxing of inclusion criteria makes study more generalized.

Data and Safety Monitoring Plan (Applicable for Clinical Trials Only):

Acceptable

- UCLA DSMB to be used.

Inclusion Plans:

- Sex/Gender: Distribution justified scientifically
- Race/Ethnicity: Distribution justified scientifically
- For NIH-Defined Phase III trials, Plans for valid design and analysis: Not Applicable
- Inclusion/Exclusion Based on Age: Distribution justified scientifically
- See comment in human subjects above.

Vertebrate Animals:
Not Applicable (No Vertebrate Animals)

Biohazards:
Not Applicable (No Biohazards)

Resource Sharing Plans:
Acceptable

Authentication of Key Biological and/or Chemical Resources:
Acceptable

Budget and Period of Support:
Recommend as Requested

THE FOLLOWING SECTIONS WERE PREPARED BY THE SCIENTIFIC REVIEW OFFICER TO SUMMARIZE THE OUTCOME OF DISCUSSIONS OF THE REVIEW COMMITTEE, OR REVIEWERS' WRITTEN CRITIQUES, ON THE FOLLOWING ISSUES:

PROTECTION OF HUMAN SUBJECTS: ACCEPTABLE

INCLUSION OF WOMEN PLAN: ACCEPTABLE

INCLUSION OF MINORITIES PLAN: ACCEPTABLE

INCLUSION ACROSS THE LIFESPAN: ACCEPTABLE

COMMITTEE BUDGET RECOMMENDATIONS: The budget was recommended as requested.

Footnotes for 1 R01 DA056287-01; PI Name: Reback, Cathy J

NIH has modified its policy regarding the receipt of resubmissions (amended applications).See Guide Notice NOT-OD-18-197 at https://grants.nih.gov/grants/guide/notice-files/NOT-OD-18-197.html. The impact/priority score is calculated after discussion of an application by averaging the overall scores (1-9) given by all voting reviewers on the committee and multiplying by 10. The criterion scores are submitted prior to the meeting by the individual reviewers assigned to an application, and are not discussed specifically at the review meeting or calculated into the overall impact score. Some applications also receive a percentile ranking. For details on the review process, see http://grants.nih.gov/grants/peer_review_process.htm#scoring.

MEETING ROSTER

Population and Public Health Approaches to HIV/AIDS Study Section
Healthcare Delivery and Methodologies Integrated Review Group
CENTER FOR SCIENTIFIC REVIEW
PPAH
11/15/2021 - 11/16/2021

Notice of NIH Policy to All Applicants: Meeting rosters are provided for information purposes only. Applicant investigators and institutional officials must not communicate directly with study section members about an application before or after the review. Failure to observe this policy will create a serious breach of integrity in the peer review process, and may lead to actions outlined in NOT-OD-14-073 at https://grants.nih.gov/grants/guide/notice-files/NOT-OD-14-073.html, NOT-OD-15-106 at https://grants.nih.gov/grants/guide/notice-files/NOT-OD-15-106.html, and NOT-OD-18-115 at https://grants.nih.gov/grants/guide/notice-files/NOT-OD-18-115.html, including removal of the application from immediate review.

CHAIRPERSON(S)

BAUERMEISTER, JOSE ARTURO, PHD
PROFESSOR
DEPARTMENT OF FAMILY AND COMMUNITY HEALTH
SCHOOL OF NURSING
UNIVERSITY OF PENNSYLVANIA
PHILADELPHIA, PA 19104

MEMBERS

ABUOGI, LISA LYNN, MD
ASSOCIATE PROFESSOR
DEPARTMENT OF PEDIATRICS
SCHOOL OF MEDICINE
UNIVERSITY OF COLORADO, DENVER
AURORA, CO 80045

AMIRKHANIAN, YURI A, PHD
PROFESSOR
DEPARTMENT OF PSYCHIATRY AND BEHAVIORAL MEDICINE
CENTER FOR AIDS INTERVENTION RESEARCH
MEDICAL COLLEGE OF WISCONSIN
MILWAUKEE, WI 53202

AUDET, CAROLYN, PHD *
ASSOCIATE PROFESSOR
DEPARTMENT OF HEALTH POLICY
UNIVERSITY OF VANDERBILT
NASHVILLE, TN 37203

BALAN, IVAN C, PHD *
RESEARCH PROFESSOR
DEPARTMENT OF BEHAVIORAL SCIENCE AND
SOCIAL MEDICINE
COLLEGE OF MEDICINE
FLORIDA STATE UNIVERSITY
TALLAHASSEE, FL 32310

BALASUBRAMANIAN, RAJI, DSC
ASSOCIATE PROFESSOR
DEPARTMENT OF BIOSTATISTICS AND EPIDEMIOLOGY
SCHOOL OF PUBLIC HEALTH AND HEALTH SCIENCES
UNIVERSITY OF MASSACHUSETTS
AMHERST, MA 01003

BARNIGHAUSEN, TILL, MD
PROFESSOR AND DIRECTOR
HEIDELBERG INSTITUTE OF GLOBAL HEALTH
FACULTY OF MEDICINE AND UNIVERSITY HOSPITAL
UNIVERSITY OF HEIDELBERG
HEIDELBERG 69120
GERMANY

BAUM, MARIANNA K, PHD *
PROFESSOR
DEPARTMENT OF DIETETICS AND NUTRITION
ROBERT STEMPEL COLLEGE OF PUBLIC HEALTH
AND SOCIAL WORK
FLORIDA INTERNATIONAL UNIVERSITY
MIAMI, FL 33199

BAUMAN, LAURIE J, PHD
PROFESSOR
DEPARTMENT OF PEDIATRICS
ALBERT EINSTEIN COLLEGE OF MEDICINE
BRONX, NY 10461

BENDAVID, ERAN, MD, MS *
ASSOCIATE PROFESSOR OF MEDICINE
DEPARTMENT OF MEDICINE
STANFORD UNIVERSITY
STANFORD, CA 94305

BIRKETT, MICHELLE, PHD
ASSISTANT PROFESSOR
DEPARTMENT OF MEDICAL SOCIAL SCIENCES
AND PREVENTIVE MEDICINE
FEINBERG SCHOOL OF MEDICINE
NORTHWESTERN UNIVERSITY
CHICAGO, IL 60611

CHRISTOPOULOS, KATERINA A, MD, MPH *
ASSOCIATE PROFESSOR
HIV/AIDS DIVISION
SAN FRANCISCO GENERAL HOSPITAL
UNIVERSITY OF CALIFORNIA, SAN FRANCISCO
SAN FRANCISCO, CA 94110

DES JARLAIS, DON C, PHD *
PROFESSOR
SCHOOL OF GLOBAL PUBLIC HEALTH
NEW YORK UNIVERSITY SCHOOL OF MEDICINE
NEW YORK, NY 10003

FRYE, VICTORIA, DRPH, MPH *
ASSOCIATE MEDICAL PROFESSOR
DEPARTMENT OF COMMUNITY HEALTH
AND SOCIAL MEDICINE
SCHOOL OF MEDICINE
THE CITY UNIVERSITY OF NEW YORK
NEW YORK, NY 10035

FUJIMOTO, KAYO, PHD
DISTINGUISHED PROFESSOR
DEPARTMENT OF HEALTH PROMOTION
AND BEHAVIORAL SCIENCES
SCHOOL OF PUBLIC HEALTH
UNIVERSITY OF TEXAS HEALTH SCIENCE CENTER
HOUSTON, TX 77030

GALARRAGA, OMAR, PHD *
ASSOCIATE PROFESSOR
DEPARTMENT OF HEALTH SERVICES,
POLICY AND PRACTICE
BROWN UNIVERSITY SCHOOL OF PUBLIC HEALTH
PROVIDENCE, RI 02912

GAMAREL, KRISTINE E, PHD *
ASSISTANT PROFESSOR
DEPARTMENT OF HEALTH BEHAVIOR
AND HEALTH EDUCATION
SCHOOL OF PUBLIC HEALTH
UNIVERSITY OF MICHIGAN
ANN ARBOR, MI 48109

GORMAN, DENNIS, PHD *
PROFESSOR
DEPARTMENT OF EPIDEMIOLOGY AND BIOSTATISTICS
TEXAS A&M SCHOOL OF PUBLIC HEALTH
COLLEGE STATION, TX 77843

HUGHES, JAMES P, PHD *
PROFESSOR
DEPARTMENT OF BIOSTATISTICS
UNIVERSITY OF WASHINGTON
SEATTLE, WA 98195

JENNESS, SAMUEL, PHD, MPH *
ASSOCIATE PROFESSOR
DEPARTMENT OF EPIDEMIOLOGY
ROLLINS SCHOOL OF PUBLIC HEALTH
EMORY UNIVERSITY
ATLANTA, GA 30030

JOSEPH DAVEY, DVORA, MPH, PHD *
ADJUNCT ASSISTANT PROFESSOR
DEPARTMENT OF EPIDEMIOLOGY
FIELDING SCHOOL OF PUBLIC HEALTH
UNIVERSITY OF CALIFORNIA LOS ANGELES
LOS ANGELES, CA 90024

KERRIGAN, DEANNA L, MPH, PHD
PROFESSOR AND CHAIR
DEPARTMENT OF PREVENTION AND COMMUNITY HEALTH
MILKEN INSTITUTE SCHOOL OF PUBLIC HEALTH
GEORGE WASHINGTON UNIVERSITY
WASHINGTON, DC 20052

KLINE, DAVID M., PHD *
ASSISTANT PROFESSOR
BIOSTATISTICS AND DATA SCIENCE
WAKE FOREST SCHOOL OF MEDICINE
WINSTON-SALEM, NC 27157

LUSENO, WINFRED K, PHD
SENIOR RESEARCH SCIENTIST
PACIFIC INSTITUTE FOR RESEARCH AND EVALUATION
CHAPEL HILL, NC 27514

MCCLELLAND, RAYMOND SCOTT, MD
PROFESSOR
DEPARTMENTS OF MEDICINE, EPIDEMIOLOGY,
AND GLOBAL HEALTH
SCHOOL OF MEDICINE
UNIVERSITY OF WASHINGTON
SEATTLE, WA 98104

NIJHAWAN, ANK ELISABETH, MD, MPH *
ASSOCIATE PROFESSOR
INTERNAL MEDICINE, DIVISION OF INFECTIOUS DISEASES
UT SOUTHWESTERN MEDICAL CENTER
DALLAS, TX 75390

NOSYK, BOHDAN, PHD *
ASSOCIATE PROFESSOR AND ENDOWED CHAIR
ECONOMICS OF HIV/AIDS
FACULTY OF HEALTH SCIENCES
SIMON FRASER UNIVERSITY AND
BC CENTRE FOR EXCELLENCE IN HIV/AIDS
VANCOUVER, BC V6Z1Y6
CANADA

OSTERMANN, JAN, PHD *
ASSOCIATE PROFESSOR
DEPARTMENT OF HEALTH SERVICES POLICY
AND MANAGEMENT
ARNOLD SCHOOL OF PUBLIC HEALTH
UNIVERSITY OF SOUTH CAROLINA
COLUMBIA, SC 29208

OUTLAW, ANGULIQUE Y, PHD *
ASSOCIATE PROFESSOR
DEPARTMENT OF FAMILY MEDICINE AND
PUBLIC HEALTH SCIENCES
SCHOOL OF MEDICINE
WAYNE STATE UNIVERSITY
DETROIT, MI 48202

OWORA, ARTHUR HAMIE, PHD *
ASSISTANT PROFESSOR
SCHOOL OF PUBLIC HEALTH
INDIANA UNIVERSITY BLOOMINGTON
BLOOMINGTON, IN 47401

PHO, MAI TUYET, MD, MPH
ASSOCIATE PROFESSOR
DEPARTMENT OF MEDICINE
SECTION OF INFECTIOUS DISEASES AND GLOBAL HEALTH
UNIVERSITY OF CHICAGO MEDICAL CENTER
CHICAGO, IL 60637

ROSENBERG, NORA, PHD *
ASSISTANT PROFESSOR
DEPARTMENT OF HEALTH BEHAVIOR
GILLINGS SCHOOL OF GLOBAL HEALTH
UNIVERSITY OF NORTH CAROLINA
CHAPEL HILL, NC 27599

SALOMON, JOSHUA A, PHD *
PROFESSOR OF HEALTH POLICY
DEPARTMENT OF HEALTH POLICY, SCHOOL OF MEDICINE
CENTER FOR HEALTH POLICY, FREEMAN SPOGLI
INSTITUTE FOR INTERNATIONAL STUDIES
STANFORD UNIVERSITY
STANFORD, CA 94305

SUNDARARAJAN, RADHIKA LU, MD, PHD *
ASSISTANT PROFESSOR
EMERGENCY MEDICINE
WEILL CORNELL MEDICINE
NEW YORK, NY 10065

WITTE, SUSAN S, PHD
PROFESSOR
SCHOOL OF SOCIAL WORK
COLUMBIA UNIVERSITY
NEW YORK, NY 10027

YOUNG, APRIL MARIE, MPH, PHD
ASSOCIATE PROFESSOR
DEPARTMENT OF EPIDEMIOLOGY
COLLEGE OF PUBLIC HEALTH
UNIVERSITY OF KENTUCKY
LEXINGTON, KY 40536

SCIENTIFIC REVIEW OFFICER

GUERRIER, JOSE H, PHD
SCIENTIFIC REVIEW OFFICER
CENTER FOR SCIENTIFIC REVIEW
NATIONAL INSTITUTES OF HEALTH
BETHESDA, MD 20892

EXTRAMURAL SUPPORT ASSISTANT

CORONADO, GISSELL DEL CARMEN
LEAD EXTRAMURAL SUPPORT ASSISTANT
CENTER FOR SCIENTIFIC REVIEW
NATIONAL INSTITUTE OF HEALTH
BETHESDA, MD 20892

* Temporary Member. For grant applications, temporary members may participate in the entire meeting or may review only selected applications as needed.

Consultants are required to absent themselves from the room during the review of any application if their presence would constitute or appear to constitute a conflict of interest.
